# Ultrathin Tunable Lens Based on Boundary Tension Effect

**DOI:** 10.3390/s19184018

**Published:** 2019-09-18

**Authors:** Ao Yang, Jie Cao, Fanghua Zhang, Yang Cheng, Qun Hao

**Affiliations:** School of optics and photonics, Beijing Institute of Technology, Key Laboratory of Biomimetic Robots and Systems, Ministry of Education, Beijing 100081, China; yangao293@126.com (A.Y.); zfh135@163.com (F.Z.); yangcheng2007@163.com (Y.C.)

**Keywords:** ultrathin tunable lens, boundary tension effect, optical imaging

## Abstract

Solid and liquid lenses are commonly used in optical design. Such lenses have suitable thicknesses due to their working principle and processing mode. Thus, zoom optical systems comprising solid and liquid lenses are extremely large. This work presents a new ultrathin tunable lens (UTL) comprising two liquid film lenses (LFLs) obtained through aspheric deformation and produced from the surface of a micro-liquid under gravity and boundary tension. The UTL can flexibly change focal lengths between positive and negative lenses when the device thickness is merely 2.15 mm. The proposed lens has the advantages of small volume, light weight, simple fabrication, and independence from external force during zooming. This research makes up for the drawback that traditional solid and liquid lenses cannot further reduce their thicknesses. The proposed UTL provides a new lens form and fabrication method, and can be used to replace solid and liquid lenses for designing miniature zoom optical systems.

## 1. Introduction

Micro-optical systems have become a research hotspot in the field of optical design and manufacturing [1,2,3]. However, the shape of traditional solid optical elements is hardly changed after processing. Meanwhile, the central and edge thicknesses of negative and positive lenses must respectively have certain numerical values to ensure the necessary strength of solid-state optical elements and keep them from being easily deformed or damaged in processing. Therefore, this kind of lens cannot easily meet the small structure and large zoom range in modern optical system design [4]. Some scholars have provided new ideas and methods for manufacturing ultrathin lenses [5,6]. Compared with a traditional zoom system, the zoom system made of the metasurface and microelectromechanical system has a smaller volume and larger tunability [7,8]. These methods have the advantages of small volume and variable focal length when manufacturing an optical system. However, this lens type has the disadvantages of high manufacturing costs and complex technology, making it difficult to fulfill the required full-field-of-view clear imaging in large zoom ranges.

Liquid lenses, which are light and enable changing of the focal length without mechanical movement [9,10], have been considered an effective alternative to traditional solid optical elements. These lenses are also widely used to design various optical systems, such as 3D displays [11], zoom systems [12,13,14], microscopes [15,16,17], endoscopes [18], and bionic human eyes [19]. However, electrowetting liquid lenses [16,20,21,22] and membrane liquid lenses [23,24,25] require suitable liquid thickness [26]. Thus, further reducing the lens volume is difficult. Moreover, the manufacturing technology of this kind of lens is complex and costly.

A novel method for reducing the volume and costs of solid optical elements and liquid lenses by using the curved surface of a micro-liquid under boundary tension to create lenses is proposed to address the aforementioned issues. With the effect of boundary tension, a small volume of liquid can form a thin curved surface that resembles a liquid film. The liquid film surface (LFS) will produce spherical deformation under the influence of gravity and boundary tension [24,27]. The curvature variation of the refracting interface changes the focus. Given this characteristic, liquid films can be used to replace solid and liquid materials in producing lenses, thereby reducing the volume and processing costs of lenses while increasing zoom ability.

## 2. Liquid Surface Deformation Analysis

### 2.1. Lens Design

**Figure 1:**
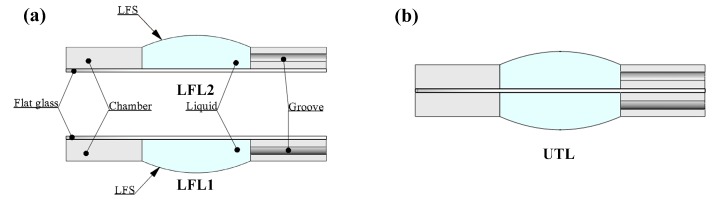
(**a**) Construction of the LFL, consisting of three main parts: chamber, flat glass, and liquid. (**b**) The cross-section of a fully assembled UTL.

The UTL is obtained by combining the LFL1 and LFL2, as shown in Figure 1b. LFL1 is the LFL in an inverted state, and LFL2 is the LFL in a positive state, as shown in Figure 1a. The structure of the LFL comprises a flat glass, the chamber, and the liquid, as shown in Figure 1b. The groove on the chamber can be used to control the volume of liquid in the chamber. Controlling the volume of liquid can change the profile of the LFS, thus changing the diopter of the UTL.

### 2.2. Mechanical Analysis

**Figure 2:**
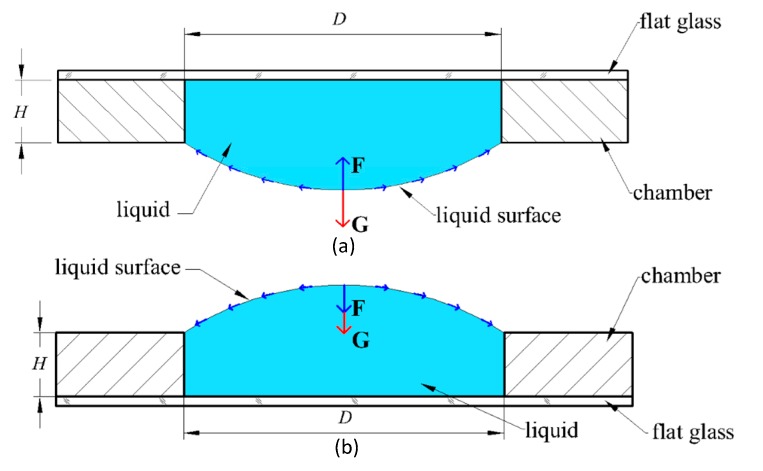
The force and deformation of the LFL. (**a**) inverted state, (**b**) positive state. D is the aperture of the chamber. H is the thickness of the chamber.

When a small amount of liquid is affected by boundary tension, the surface of the liquid becomes similar to an elastic film under uniform force, thereby tending to reduce the volume of the liquid and bending the LFS. The force and deformation of the LFL in the inverted state are shown in Figure 2a, and those for the positive state are in Figure 2b.

In [28], a second-order, second-degree equation of force and deformation on a droplet surface is written as Equation (1). This formula can be used to analyze the surface profile of the droplet in Figure 2.

(1) $$\frac{\frac{d^{2}z}{dx^{2}}}{{\left[1+{\left(\frac{dz}{dx}\right)}^{2}\right]}^{\frac{3}{2}}}+\frac{\frac{dz}{dx}}{x{\left[1+{\left(\frac{dz}{dx}\right)}^{2}\right]}^{\frac{1}{2}}}=\frac{2}{R}\pm \frac{g\rho z}{\gamma }$$

where *x* is the horizontal distance to the axis of the drop, *z* is the vertical coordinate measured away from an origin placed at the point where the axis of rotation cuts the surface of the drop, *R* is the curvature radius of the drop at the origin, *γ* is the boundary tension, *ρ* is the density of fluid, and *g* is the acceleration of gravity.

**Figure 3:**
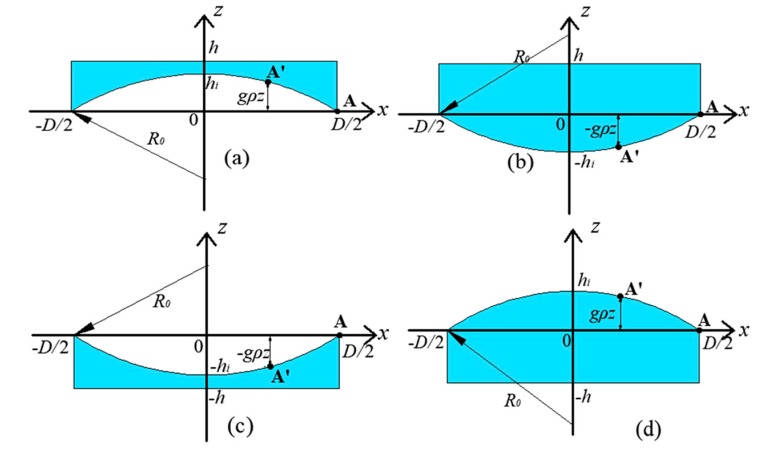
Deformation model, (**a**) concave LFS in the inverted state, (**b**) convex LFS in the inverted state, (**c**) concave LFS in the positive state, and (**d**) convex LFS in the positive state.

The deformation model of concave LFS and convex LFS are shown in Figure 3. The boundary of the cavity is set as the x-axis, and the intersection point A of the LFS and the x-axis is selected as the origin. Any point A′ on the LFS and the origin A shows a difference in gravity. If the point A′ is higher than the origin A, then the gravity difference is positive, as shown in Figure 3a,d. If the point A′ is lower than the origin A, then the gravity difference is negative, as shown in Figure 3b,c.

### 2.3. Numerical Solution of the Lfs Profile 

The relationship between the ordinate *z* and abscissa *x* of the points on the LFS cannot be solved by Equation (1) because the radius of curvature *R* at the origin is unknown. Therefore, the solution formula of the volume of liquid is introduced.

(2) $$V_{i}=\pi {\left(\frac{D}{2}\right)}^{2}h\pm |\int_{0}^{\pm h_{i}}\pi x^{2}dx|$$

where, *V*_i_ and *h*_i_ are the volume of liquid and the sag of LFS, respectively, both of which are obtained by the *i*th solution. The positive and negative signs in the formula correspond to the volume of liquid larger than the cavity volume and smaller than the cavity volume, respectively.

The numerical solution of the LFS profile can be obtained by combining Equation (1) and Equation (2). The solution process is as follows.

1. With Equation (1), the corresponding relationship between ordinate *z* and abscissa *x* can be solved by giving a non-zero origin curvature *R*_i_ with ODE45, which is a numerical solution function of the ordinary differential equation in MATLAB.
2. With the aid of the trapezoidal numerical integration formula (TRAPZ) in MATLAB, the integral volume of the area surrounded by the LFS can be conducted for the numerical solution of the LFS profile, which is obtained in the first step. The volume of liquid *V*_i_ can then be calculated by Equation (2).
3. The volume of liquid *V*_i_ obtained by the second step is compared with the given volume of liquid *V*_0_, and their difference is calculated as *m*_i_ = |(*V*_i_ − *V*_0_)/*V*_0_|. Meanwhile, the volume ratio can be calculated as *w*_i_ = *V*_i_/*V*_0_.
4. If *m*_i_ < 10^−4^, then the calculated volume of liquid *V*_i_ is consistent with the given volume of liquid *V*_0_ and the output relationship between ordinate *z* and abscissa *x*.
5. If *m*_i_ > 10^−4^, then the calculated volume of liquid *V*_i_ is inconsistent with the given volume of liquid *V*_0_. The origin curvature is therefore corrected as *R*_i+1_ = *w*_i_ × *R*_i_. *R*_i+1_ is substituted into the first step, and Steps (1)–(4) are repeated until the relationship between ordinate *z* and abscissa *x* of the LFS profile satisfies the requirements.

**Figure 4:**
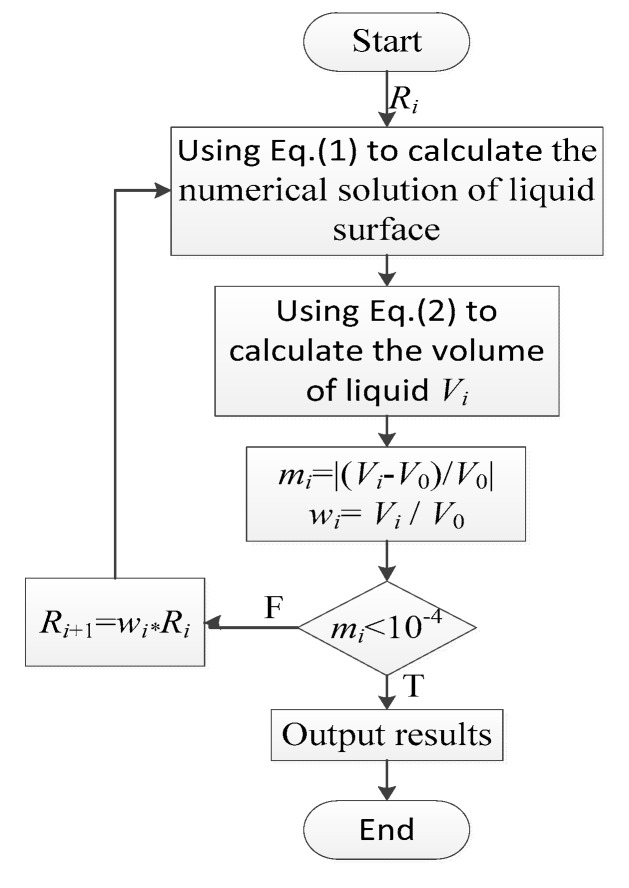
Program flow diagram.

The program flow diagram is shown in Figure 4.

**Figure 5:**
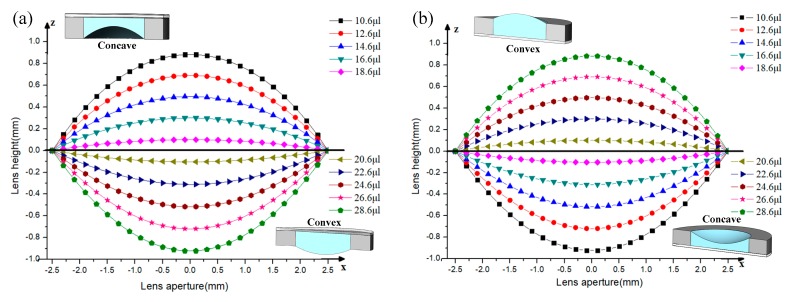
The LFS profiles of the LFL. (**a**) Inverted state. (**b**) Positive state.

For calculation, we specify some parameters of LFL. The cavity diameter *D* is 5 mm. Water is used as the liquid material to solve the deformation of the LFS profile. The boundary tension of water *γ* is 72 mN/m, and the density of water *ρ* is 1 g/mL. The numerical solution of the LFS profile with different volumes of liquid can be obtained by using the above steps. The calculated profiles of LFS are illustrated in Figure 5.

## 3. Optical Simulation of the Ultrathin Tunable Lens (UTL)

**Figure 6:**
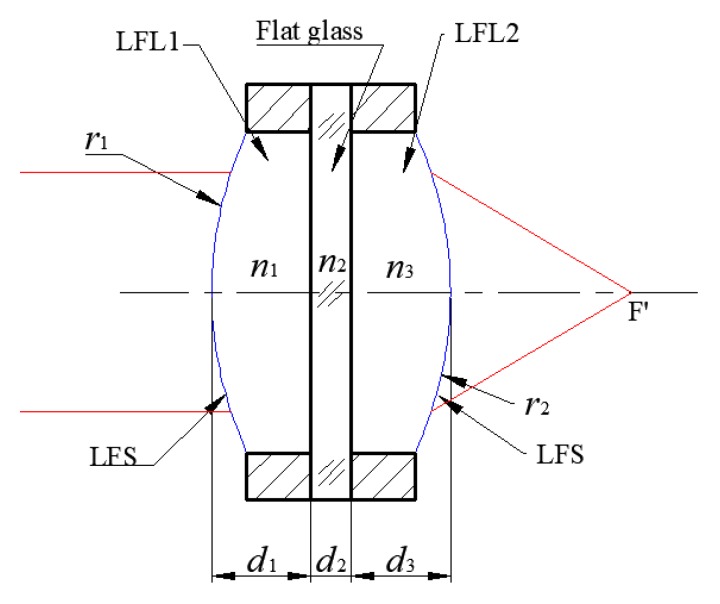
The structure of the UTL. *r*_1_ and *r*_2_ are the curvatures of two LFSs, respectively. *n*_1_ is the index of refraction of LFL1, and *n*_3_ is the index of refraction of LFL2. *n*_2_ is the index of refraction of the flat glass. *d*_1_ and *d*_3_ are the thicknesses of the LFL1 and LFL2, respectively. *d*_2_ is the thickness of the flat glass.

The UTL can be regarded as a lens consisting of three elements, as shown in Figure 6.

Given that the profile of the deformed LFS is non-spherical, an even aspheric surface can be used to fit the profile of the deformed LFS. The expression of an even aspheric surface can be given as [29]

(3) $$z=\frac{cx^{2}}{1+\sqrt{1-(1+k)c^{2}x^{2}}}+A_{1}x^{4}+A_{2}x^{6}+A_{3}x^{8}+A_{4}x^{10}$$

where *c* is the vertex curvature of the LFS profile, *k* is the vertex conic constant (which is assumed as −1 (paraboloid)), and *A*_1_–*A*_4_ are the aspheric surface coefficient.

The numerical solution of the LFS profile, which was obtained in Section 2, can be fitted with the even aspheric surface by using Equation (3). Meanwhile, the curvature radius *r* of the LFS profile can be calculated as *r* = 1/*c*.

The formula for calculating the focal length of the UTL can be derived from the formula of refraction between the spherical object–image relationship and the formula of the turning surface, which are expressed as

(4) $$\{\begin{array}{c} \frac{n_{k}^{\prime }}{l_{k}^{\prime }}-\frac{n_{k}}{l_{k}}=\frac{n_{k}^{\prime }-n_{k}}{r_{k}}\\ n_{k+1}=n_{k}^{\prime }\\ l_{k+1}=l_{k}^{\prime }-d_{k}\\ f^{\prime }=\frac{l_{1}^{\prime }l_{2}^{\prime }\cdot \cdot \cdot l_{k}^{\prime }}{l_{2}l_{3}\cdot \cdot \cdot l_{k}} \end{array}$$

where *n_k_* and *l_k_* are the index of refraction and the object distance in the object plane, respectively, and *n’_k_* and *l’_k_* are the corresponding values in the image space. *r_k_* is the radius of curvature of the *k*th surface, and *d_k_* is the distance between the *k*th surface and the (*k* + 1)th surface.

When the object is located at infinite distance, it is *l*_1_ = ∞. According to Equation (4), the expression of focal length of the UTL can be calculated as

(5) $$f^{\prime }=\frac{n_{1}r_{1}r_{2}}{n_{1}(n_{1}-1)(r_{2}-r_{1})+{\left(n_{1}-1\right)}^{2}(d_{1}+\frac{n_{1}}{n_{2}}d_{2}+d_{3})}$$

**Figure 7:**
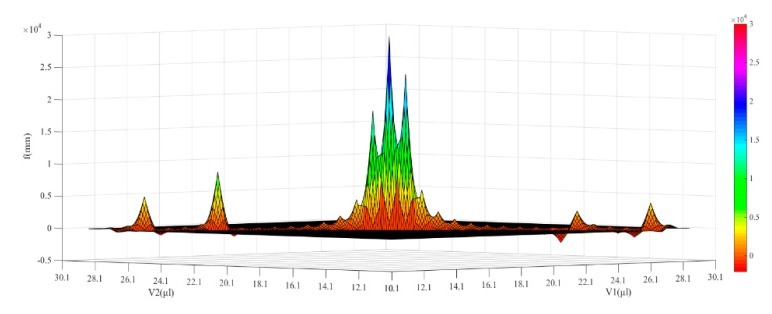
The optical simulation layouts of the UTL with different focal lengths.

In this study, the optical parameters of the UTL are shown in Table 1. The relationship between the focal length of the UTL and the injection volume of two LFLs can be obtained by substituting the radius values (*r*_1_ and *r*_2_) of LFL1 and LFL2 from different volumes fit into Equation (3) and the parameters in Table 1 into Equation (5). The resulting calculation is shown in Figure 7.

## 4. Experiment and Result Discussion

**Figure 8:**
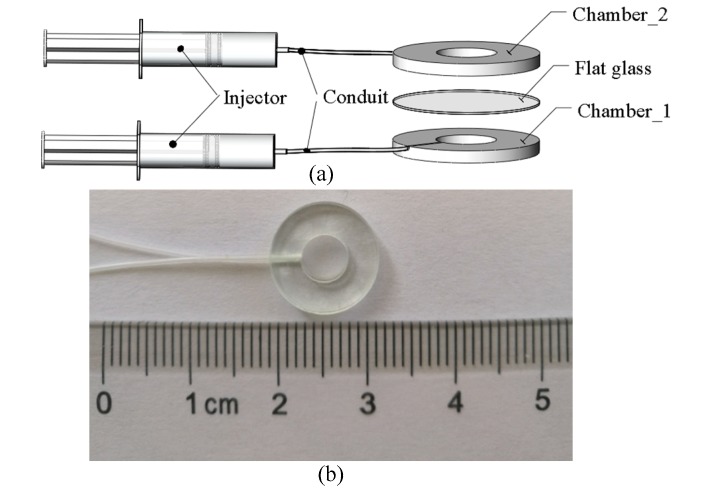
(**a**) Schematic showing the UTL construction. (**b**) Photograph of the UTL.

The fabricated UTL is shown in Figure 8. The material of the chamber is glass. The aperture and external diameter of the chamber are 5 and 12 mm, respectively. The thickness of the chamber is 1 mm. A groove for liquid flow is machined on the bottom. The groove is connected with an injector (50 μL) through the rubber conduit, and the gap at the joint is sealed with UV glue. The top chamber, bottom chamber, and flat glass are bonded by UV glue.

**Figure 9:**
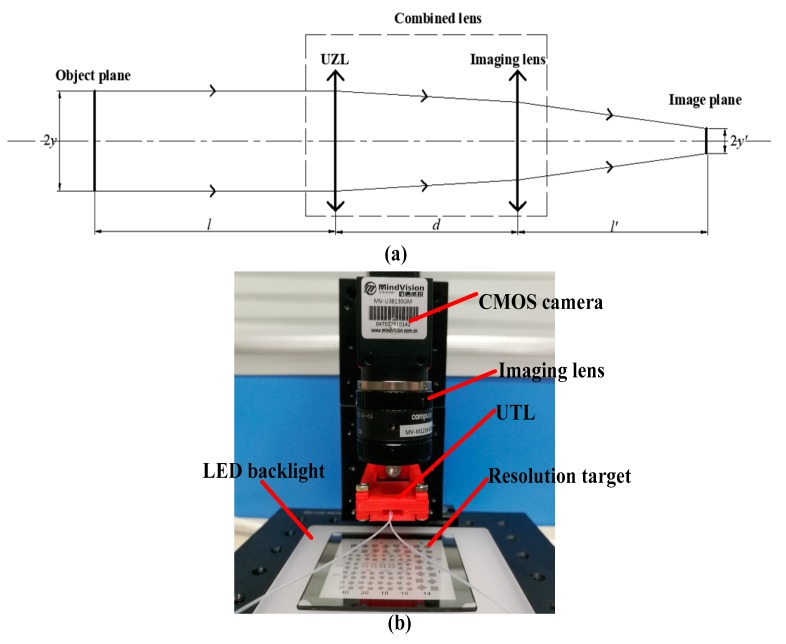
(**a**) Experimental principle and (**b**) experimental device.

The experimental structure is shown in Figure 9. A resolution target is selected as the object and placed 20 mm from the UTL. The focal length of the UTL can be changed from a negative lens to a positive lens. Therefore, an imaging lens (MV-M1214-MP2-D, from Computer, Japan) with a focal length of 12 mm is combined with the UTL to form a combined lens that resembles the object plate. The image is received by a black and white camera (MV-U3B130GM, from MindVision, Shenszhen, China) with a pixel size of 3.75 μm and sensor size of 1/3 inch. The experimental process is as follows.

**Figure 10:**
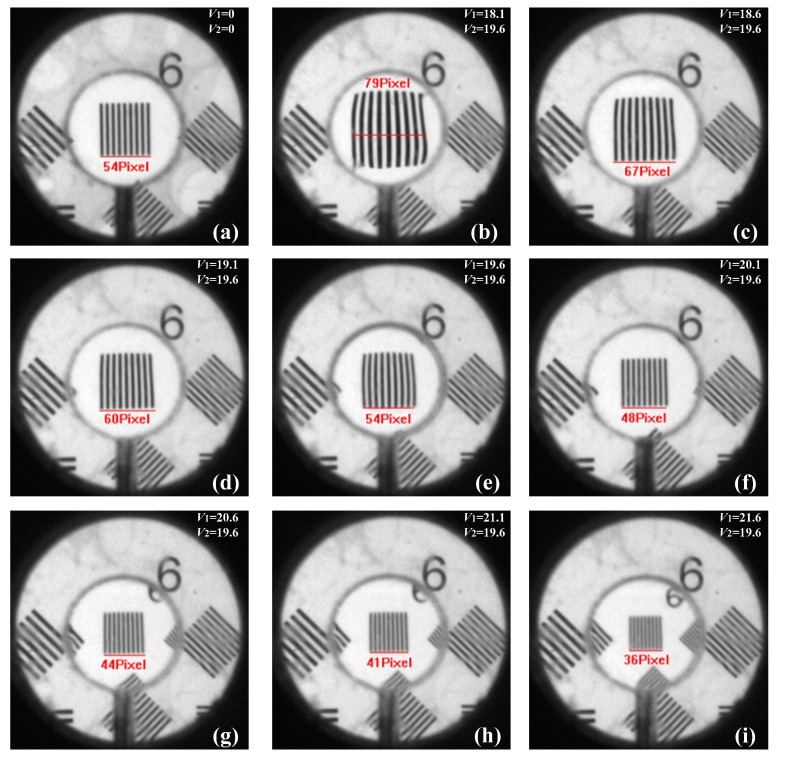
Captured images using a resolution target. (**a**) When no water is injected into the UTL, the image height of the resolution target *y′*_0_ is obtained. (**b**–**i**) Changing the volume of injected water in LFL1 to change the focal length of the UTL.

1. The image height of the resolution target *y′*_0_ is obtained when no liquid is injected into the UTL, as shown in Figure 10a.
2. To form a flat lens, 19.6 microbubbles are injected into LFL2.
3. The zoom of the UTL is controlled by adjusting the volume of liquid injected into LFL1. Simultaneously, with the adjustment of the distance, a clear image and its height *y′*_n_ of the *n*th zoom can be obtained by the camera. The imaging results of the UTL with different focal lengths are shown in Figure 10b–i.

The focal length of the UTL at the *n*th zoom can be calculated by

(6) $$f_{n}^{\prime }=\frac{l}{\frac{y_{0}^{\prime }}{y_{n}^{\prime }}-1},$$

where *f′*_n_ is the focal length of the UTL at the *n*th zoom, *l* is the distance between the object plane and the UTL, *y′*_0_ is the image height when *f′* = ∞, and *y′*_n_ is the image height at different focal lengths of the UTL.

**Figure 11:**
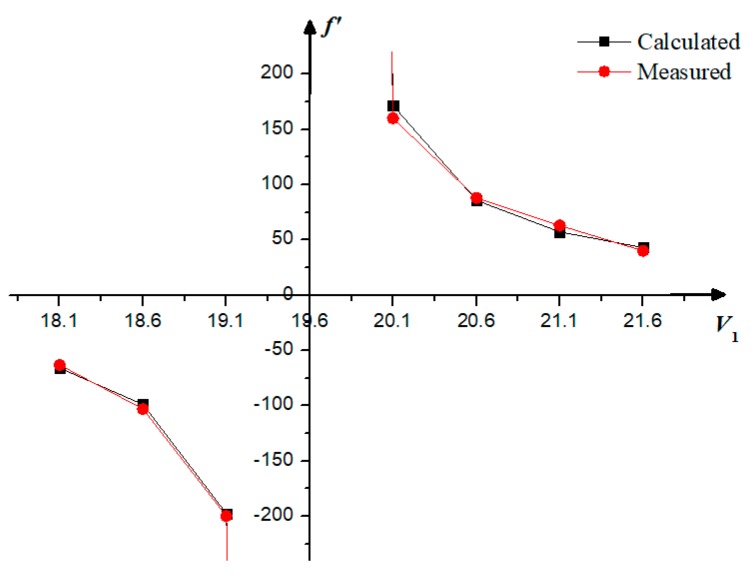
The focal length of the UTL comparison among calculated and measured values.

The measured and calculated results of the focal length of the UTL are shown in Table 2, and the focal lengths of the UTL comparison among calculated and measured values are shown in Figure 11. In the experiment, the UTL had a very large zoom range when zoomed from the positive to negative lens. The measured results of focal length are consistent with the calculated values. Because the lens was not designed to eliminate aberration, the result has obvious distortion, as shown in Figure 10b,c.

The zoom results in Figure 10 show that the UTL has certain feasibility in practical applications. However, because the surface deformation of the UTL is based on gravity and surface tension, the UTL can only be applied in cases where gravity is collinear with the optical axis of the lens. In addition, the evaporation of liquids can be avoided by choosing different kinds of liquids (such as silicone oil, polydimethylsiloxane) or by using lens encapsulation. At the same time, the influence of temperature on zoom accuracy can be compensated for by establishing a zoom compensation system for the UTL. These methods can further improve the feasibility of a liquid lens in practical applications.

The liquid is injected into the chamber by an injector and the speed of tunability in Figure 10 is slow. Therefore, in the future research, in order to improve the zoom speed of the UTL, we plan to use a piezoelectric crystal instead of the injector as the liquid actuator, so as to improve the zoom speed and precision of the UTL.

## 5. Conclusions

This paper studies the surface deformation of a micro-liquid under boundary tension. This research makes up for the drawback that traditional solid and liquid lenses cannot further reduce their thicknesses. The UTL proposed in this paper only relies on the effect of boundary tension to produce surface bending. Consequently, the focal length between positive and negative lenses can be flexibly changed while the device thickness remains only 2.15 mm. Compared with solid and liquid lenses, the UTL has the advantages of small volume, simple fabrication, and independence from complex institutions during zooming due to its new surface-forming method. Therefore, the UTL can be used instead of solid or liquid lenses to design optical zoom systems, reduce their volume and weight, and improve their zoom ability and accuracy. In future work, improvement of the structure and manufacturing technology of the UTL will be continued to enhance its imaging effect, and the combination zoom and aberration correction of several UTLs will be further discussed.

## Figures and Tables

**Table 1:** The optical parameters of the UTL.

	LFL1	Flat Glass	LFL2
Material	water	K9	water
Index of refraction	*n*_1_ = 1.333	*n*_2_ = 1.516	*n*_3_ = 1.333
Thickness	*d*_1_ = 1 − *h_i_*	*d*_2_ = 0.15	*d*_3_ = 1 + *h_i_*

**Table 2:** Comparisons between the measured and calculated results.

*V*_1_ (μL)	Calculated (mm)	Measured (mm)
18.1	−66.06	−63.2
18.6	−98.93	−103.07
19.1	−197.68	−200
19.6	∞	∞
20.1	171.53	160
20.6	85.83	88
21.1	57.29	63.07
21.6	43.03	40
